# Interactions between HIV-1 Reverse Transcriptase and the Downstream Template Strand in Stable Complexes with Primer-Template

**DOI:** 10.1371/journal.pone.0003561

**Published:** 2008-10-30

**Authors:** Wiriya Rutvisuttinunt, Peter R. Meyer, Walter A. Scott

**Affiliations:** Department of Biochemistry and Molecular Biology, University of Miami Miller School of Medicine, Miami, Florida, United States of America; The Scripps Research Institute, United States of America

## Abstract

**Background:**

Human immunodeficiency virus type 1 reverse transcriptase (HIV-1 RT) forms stable ternary complexes in which RT is bound tightly at fixed positions on the primer-template (P/T). We have probed downstream interactions between RT and the template strand in the complex containing the incoming dNTP (+1 dNTP•RT•P/T complex) and in the complex containing the pyrophosphate analog, foscarnet (foscarnet•RT•P/T complex).

**Methods and Results:**

UV-induced cross-linking between RT and the DNA template strand was most efficient when a bromodeoxyuridine residue was placed in the +2 position (the first template position downstream from the incoming dNTP). Furthermore, formation of the +1 dNTP•RT•P/T complex on a biotin-containing template inhibited binding of streptavidin when biotin was in the +2 position on the template but not when the biotin was in the +3 position. Streptavidin pre-bound to a biotin residue in the template caused RT to stall two to three nucleotides upstream from the biotin residue. The downstream border of the complex formed by the stalled RT was mapped by digestion with exonuclease RecJ_F_. UV-induced cross-linking of the complex formed by the pyrophosphate analog, foscarnet, with RT and P/T occurred preferentially with bromodeoxyuridine in the +1 position on the template in keeping with the location of RT one base upstream in the foscarnet•RT•P/T complex (i.e., in the pre-translocation position).

**Conclusions:**

For +1 dNTP•RT•P/T and foscarnet•RT•P/T stable complexes, tight interactions were observed between RT and the first unpaired template nucleotide following the bound dNTP or the primer terminus, respectively.

## Introduction

Human immunodeficiency virus type I reverse transcriptase (HIV-1 RT) is essential for replication of the viral genomic RNA into a double-stranded DNA intermediate that is subsequently integrated into the host genome. Various complexes between HIV-1 RT and primer-template (P/T) have been characterized as a result of investigations into the role of RT in viral DNA synthesis [1]–[8]. Comparisons between the crystal structures of the binary (RT•P/T) [1], [5], [9] and ternary complexes (+1 dNTP•RT•P/T)[4], [10] show that binding of the next complementary dNTP results in a conformational change in which the enzyme closes in on the P/T and the “fingers” subdomain moves closer to the downstream (single-stranded) portion of the template. The resulting ternary complex is stable to dissociation by competitor P/T or by heparin, in contrast to the binary complex, which is readily dissociated under the same conditions [3], [11], [12]. Discrete barriers to exonuclease digestion were observed for the +1 dNTP•RT•P/T complex, which provide information about the upstream and downstream borders of the complex [8]. The pyrophosphate analog, foscarnet (phosphonoformic acid, PFA), also induces formation of a stable complex (foscarnet•RT•P/T complex) positioned approximately one nucleotide upstream from the +1 dNTP•RT•P/T complex [7], [8], [13], [14].

Nuclease protection experiments [2], [8] and crystallographic studies on the +1 dNTP•RT•P/T complex [4] have shown that the RT makes contacts with the downstream portion of the template. With P/Ts having very short single-stranded extensions, the formation of the +1 complex is greatly reduced [3], [15]. The functional importance of interactions between RT and downstream portions of the template was first reported by Boyer et al. [16], who showed that the *in vitro* sensitivity of HIV-1 RT to 2′,3′-dideoxyinosine triphosphate or 2′,3′-dideoxyadenosine triphosphate required a template overhang of at least 3 or 4 nucleotides. In addition, Winshell and Champoux [17] showed that RT could melt a downstream duplex structure, with the melted region extending two bases in front of the primer terminus. More recently, Dash et al. [18] have characterized the stalling patterns of HIV-1 RT as it approaches various lesions in the template strand. The presence of a nucleotide analog containing a conformationally locked cyclohexane ring in place of the pentose sugar caused RT to stall two nucleotides upstream from the lesion, with minor stalling observed three and four nucleotides upstream. These results indicate that HIV-1 RT makes contacts with the single-stranded portion of the template at least two bases downstream from the primer terminus.

We show that UV photo-cross-linking to a bromodeoxyuridine (BrdU) residue located at the +2 position on the template is favored by the presence of the next complementary dNTP and that cross-linking to the +1 position is favored by the presence of foscarnet. Throughout this paper, +1 refers to the first nucleotide and +2 to the second nucleotide downstream from the primer terminus. The +2 position is also the first template position downstream from the bound dNTP in the +1 dNTP•RT•P/T complex. The base pair that includes the primer terminus is designated as -1. An artificial barrier created by binding streptavidin (SA) to a biotin residue in the template caused RT primer extension to stall two nucleotides upstream from the biotin residue indicating that RT protrudes at least two nucleotides beyond the end of the primer. Our results suggest that the primary downstream contacts in the +1 dNTP•RT•P/T complex are at the +2 position on the template. Contacts at the +3 position could also be detected, but contacts further downstream were not observed.

## Materials and Methods

### Oligodeoxynucleotides and Nucleotides

**Figure 1:**
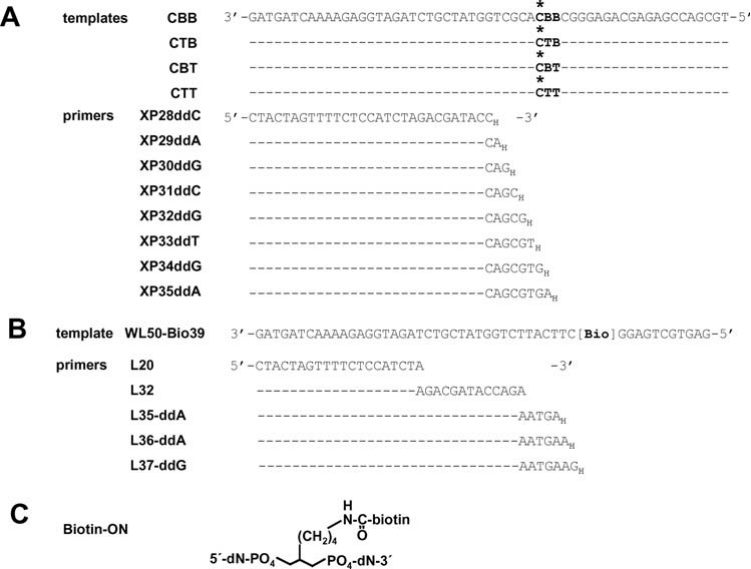
DNA primer-templates. (A) Primer-templates used for photo-cross-linking studies. “B” indicates a BrdU residue and * shows the position of an α-^32^P-labeled dC residue in the template. Dashes indicate that the sequence is the same as the line above. Subscript “_H_” indicates a dideoxynucleotide residue introduced at the 3′ end of an oligodeoxynucleotide primer. (B) Primer-templates used for SA binding and SA-biotin barrier experiments. “Bio” indicates the position of the internal Biotin-ON structure (shown in panel (C)). WL50 has the same sequence as WL50-Bio39 with the Biotin-ON structure replaced by T.

HPLC-purified WL50-Bio39 containing an internal Biotin-ON residue (shown in Figure 1C) was purchased from QIAGEN-Operon. Other synthetic oligodeoxynucleotides were purchased from Sigma Genosys and gel-purified prior to use. Unlabeled dideoxynucleoside triphosphates (ddNTPs), dNTPs and ATP were purchased from GE Healthcare. Unlabeled dNTPs were pretreated with thermostable pyrophosphatase (Roche Applied Science) as previously described [19]. [α-^32^P]dCTP and [α-^32^P]ddATP were from GE Healthcare. [γ-^32^P]ATP and [^3^H]dTTP were from PerkinElmer Life Sciences. BrdUTP was purchased from Sigma-Aldrich or from TriLink BioTechnologies. Oligodeoxynucleotides were 5′-end labeled with T4 polynucleotide kinase (New England Biolabs, Inc.) and [γ-^32^P]ATP, or 3′-end labeled with [α-^32^P]ddATP and terminal transferase (New England Biolabs, Inc.) as described by the supplier. Internal labeling of BrdU-containing templates with [α-^32^P]dCTP is described below. Unlabeled 5′-phosphorylated oligodeoxynucleotides were prepared with T4 polynucleotide kinase and unlabeled ATP.

### Preparation of Chain-terminated Primers

The unlabeled chain-terminated primers shown in Figure 1A were prepared from approximately 100 pmoles of each oligodeoxynucleotide by incubating with 20 units of terminal transferase (New England Biolabs, Inc.) and 0.7 mM of the appropriate ddNTP in a total volume of 100 µl containing 50 mM potassium acetate, 20 mM Tris-acetate, pH 7.9, 10 mM magnesium acetate, 1 mM DTT, and 0.25 mM CoCl_2_. Incubation was for 30 min at 37°C followed by phenol/chloroform extraction and ethanol precipitation.

To prepare the chain-terminated primers L35-ddA and L36-ddA shown in Figure 1B the unterminated oligodeoxynucleotides were 5′-end labeled, annealed with a 4-fold excess of WL50-Bio39 in 40 mM HEPES-HCl, pH 7.5, 60 mM KCl by heating at 90°C for 5 min and slowly cooling to room temperature, and chain-terminated by incubating with 200 nM HIV-1 RT in RB buffer (40 mM HEPES-HCl, pH 7.5, 20 mM MgCl_2_, 60 mM KCl, 1 mM DTT, 2.5% glycerol) containing 0.08 mg/ml bovine serum albumin and 100 µM of the next complementary ddNTP for 30 min at 37°C, followed by phenol/chloroform extraction and ethanol precipitation. L37-ddG was prepared from L36 by annealing to WL50-Bio39 and extending with dATP and ddGTP. The size of the extended product was confirmed by electrophoresis.

### Reverse Transcriptase

**Figure 2:**
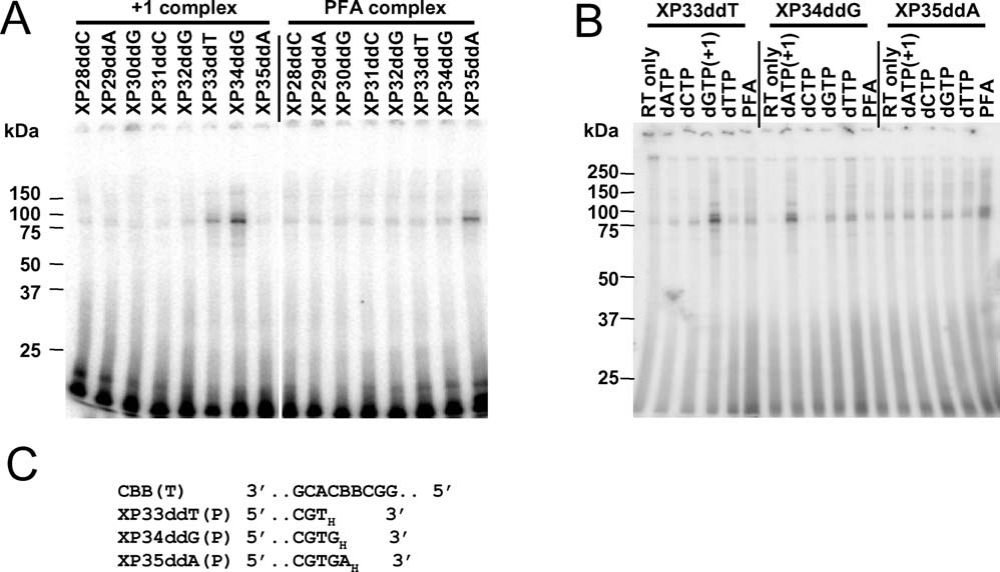
Photo-cross-linking of HIV-1 RT to template containing two adjacent BrdU residues. (A) Stable complexes were formed by incubating 40 nM HIV-1 RT, 9 nM P/T (the indicated primer annealed to CBB template (Figure 1)), and 100 µM of the +1 dNTP (+1 complex); or 200 nM HIV-1 RT, 9 nM P/T, and 3.2 mM foscarnet (PFA complex) for 10 min at 37°C followed by cooling in ice. Heparin was added and the mixture was kept in ice and exposed to UV light as described in the text. Samples were analyzed by SDS-PAGE. Positions of MW markers are indicated at the left of each panel. (B) Complexes were prepared and analyzed as in (A) except that 5 nM of each P/T was mixed with 200 nM RT only or with RT plus 100 µM of each of the indicated dNTPs or RT plus 3.2 mM foscarnet. “+1” indicates the dNTP complementary to the first unpaired template nucleotide following the primer terminus. (C) Portions of the CBB template (T) sequence and selected primer (P) sequences are shown. “B” indicates BrdU. Subscript “_H_” denotes a dideoxynucleotide residue. The experiment in (A) was repeated with heterodimer RT obtained from Worthington Biochemical Corp. with similar results.

His-tagged wild type HIV-1 RT was expressed from pKRT2, provided by R. D'Aquila and W.C. Summers [20] through the AIDS Research and Reference Reagent Program, Division of AIDS, NIAID, and modified to add an N-terminal polyhistidine tag as previously described [21]. The RT coding sequence was derived from the BH10 clone of HIV-1 [20], expressed in JM109 *E. coli,* and purified by metal affinity chromatography (His-Bind Resin, Novagen). Specific activity was 20,000 to 23,000 units/mg where one unit corresponds to the amount of enzyme needed for incorporation of 1.0 nmole [^3^H]dTMP in 10 min at 37°C using poly(rA)/oligo(dT) as substrate [22]. This construct was expressed as p66/p66 homodimer but proteolytic cleavage of one of the p66 subunits by unidentified protease activity during purification or storage produced polypeptide about 51 kDa [23]. The p66 to p51 ratio, determined by quantifying the intensity of Coomassie Brilliant Blue stain after fractionation of the subunits on a denaturing polyacrylamide gel, indicated the presence of about 74% heterodimer and 26% homodimer RT in these experiments. The experiment in Figure 2A was also repeated with commercial p66/p51 heterodimer RT strain HXB2 lacking the polyhistidine tag (Worthington Biochemical Corp).

### Preparation of Internally ^32^P-Labeled, BrdU-Containing Templates

Labeled BrdU-containing templates were prepared essentially as described by Coman et al. [24]. To prepare CBB or CTT template (Figure 1A) 100 pmoles of oligodeoxynucleotide XT20 (5′-TGCGACCGAGAGCAGAGGGC-3′) were annealed with 400 pmoles of a scaffold oligodeoxynucleotide XL69 (5′-GTGTGTGTGTGTCTACTAGTTTTCTCCATCTAGACGATACCAGCGTGAAGCCCTCTGCTCTCGGTCGCA-3′) in 20 mM Tris-HCl, pH 7.5, 150 mM NaCl and extended with Sequenase Version 2.0 DNA polymerase (USB Corp.). Sequenase Version 2.0 (consisting of exonuclease-deficient T7 gene 5 protein and *E. coli* thioredoxin) was preincubated at a concentration of 2 units/µl in 6.5 mM Tris-HCl, pH 7.5, 3.0 mM potassium phosphate, 6.4 mM DTT, 0.08 mM EDTA, and 7.5% glycerol for 20 min on ice. XT20 extension was carried out in a volume of 200 µl with 3–7 units of Sequenase, 90 µM BrdUTP (for synthesis of CBB template) or 50 µM dTTP (for synthesis of CTT template), 50 mM Tris-HCl, pH 7.5, 20 mM MgCl_2_, 125 mM NaCl, 0.1 mg/ml BSA and 1 mM DTT at 37°C for 45 min followed by addition of 40 µCi [α-^32^P]dCTP and further incubation at 37°C for 30 min. Then 1 mM of unlabeled dCTP was added and incubation continued for 20 min. The sample was extracted with phenol/chloroform, precipitated with ethanol, resuspended in 132 mM Tris-HCl, pH 7.5, and 40 mM KCl, and annealed with 160 pmoles 5′ phosphorylated XT34 oligodeoxynucleotide (5′-ACGCTGGTATCGTCTAGATGGAGAAAACTAGTAG-3′). Ligation was carried out for 1-2 h at 25°C in a total volume of 50 µl containing 113 mM Tris-HCl, pH 7.5, 36 mM KCl, 10 mM MgCl_2_, 2 mM DTT, 2 mM ATP, 3% PEG 6000, and 2.5 µl (5000 units) of Quick T4 DNA ligase (New England Biolabs, Inc.). The sample was fractionated by electrophoresis on 10% polyacrylamide in 7 M urea and the 57-nucleotide ligation product was recovered by excision, eluted by soaking overnight in 0.5 M ammonium acetate, 10 mM magnesium acetate, 1 mM EDTA, 0.1% SDS, pH 8.0, and recovered by ethanol precipitation.

Template CBT was prepared as described above except that XT21 (5′-TGCGACCGAGAGCAGAGGGCT-3′) was annealed with XL69 and extended with BrdUTP and [α-^32^P]dCTP followed by ligation with 5′-phosphorylated XT34. Template CTB was prepared by annealing synthetic oligodeoxynucleotide XT20BT (5′-TGCGACCGAGAGCAGAGGGC[BrdU]T-3′) with XL69, extending with [α-^32^P]dCTP and ligating with 5′-phosphorylated XT34.

### Photo-Cross-Linking Reactions

CBB, CTT, CTB or CBT template was annealed with a 4-fold excess of each of the eight chain-terminated primers shown in Figure 1A and 5 to 20 nM P/T was incubated with 4 to 80-fold excess of HIV-1 RT, without or with 100 µM dNTP or with 3.2 mM foscarnet, in 10 µl RB buffer for 5–10 min at 37°C, then cooled in ice for 5 min. P/T and RT concentrations are given in the figure legends. Foscarnet was purchased as 80 mM Foscavir intravenous solution from AstraZeneca. The reaction mixture was transferred to an ice-cold 96-well Costar brand UV transparent plate (Corning, Inc., Life Sciences) containing heparin solution (final concentration 2.3 mU/ml) and exposed to 1 Joule UV light from a GS Gene Linker (BioRad Labs.) Samples were heated (4 min at 90°C) in the presence of 1% SDS, 1% β-mercaptoethanol, 0.03% bromphenol blue, 10% sucrose, and 0.05 M Tris-HCl, pH 6.8, and fractionated by SDS-PAGE on a 10% polyacrylamide gel.

### Electrophoretic Mobility Shift Assays (EMSA) for dNTP-Induced Stable Complex Formation and for Detection of Streptavidin (SA) Bound to Biotin-Containing DNA

EMSA assay for heparin-resistant stable complex formation on the 5 nM 5′-labeled chain-terminated primers annealed to biotin-containing WL50-Bio39 template (Figure 1B) was carried out as previously described [8] except that HIV-1 RT concentration was 100 nM. Additional EMSA assays were carried out to determine if prior binding of dNTP could prevent the binding of SA (Sigma Aldrich) to the template biotin residue [25]. After 10 min incubation at 37°C with dNTP, the reaction mixtures were cooled on ice for 5 min and SA was added to a final concentration of 50 to 250 nM and incubated at 37°C for 2–5 min. Then free biotin (Sigma Aldrich) was added to a final concentration of 0.6 to 6.0 µM as indicated and the incubation was continued at 37°C for 5 min to trap excess SA. RT was dissociated by addition of an equal volume of loading dye-urea-TTE (16 M urea, 180 mM Tris-HCl, pH 9.0, 60 mM taurine, 0.5 mM EDTA, 0.25% (w/v) bromphenol blue, 0.25% (w/v) xylene cyanol) with 0.5% SDS prior to electrophoresis. These conditions dissociate RT from the P/T but do not dissociate SA bound to the biotin residue in the template. SA-DNA complexes were separated from free DNA by electrophoresis on a 6% native polyacrylamide gel in Tris-taurine buffer (90 mM Tris, 30 mM taurine, pH 7.5).

### Primer Extension

Five nM 5′-^32^P-labeled L32 primer or L20 primer annealed with WL50-Bio39 template was incubated without or with 100 nM SA in RB buffer and 0.08 mg/ml BSA at 37°C for 2-3 min. Then HIV-1 RT (200 nM) was added with 100 µM each of the four dNTPs and 6 µM free biotin, and the mixture was incubated at 37°C for 10 to 45 min to allow primer extension. An equal volume of loading dye-urea-TTE was added and products were fractionated by electrophoresis through a 20% denaturing polyacrylamide gel.

### Detection of Barriers to RecJ_F_ Exonuclease Digestion

WL50-Bio39 template was 3′-end labeled with [α-^32^P]ddATP and terminal transferase and annealed to unlabeled L32 primer. Five nM P/T was incubated with 50 nM SA in RB buffer and 0.08 mg/ml BSA at 37°C for 5 min, and cooled in ice. An aliquot was taken for digestion with RecJ_F_ exonuclease (New England Biolabs, Inc.) at 1 U per µl of reaction mixture and incubated at 37°C, sampling at various times as previously described [8]. HIV-1 RT (12.5 nM) and 100 µM each of the four dNTPs was added to an additional aliquot and incubated for 10 min at 37°C, followed by addition of RecJ_F_ and additional incubation as indicated above. Incubation was terminated by addition of an equal volume of loading dye-urea-TTE and electrophoresis on a 20% denaturing polyacrylamide gel.

## Results

### Cross-linking between RT and BrdU-containing template DNA in the presence of +1 dNTP

A DNA template containing two adjacent BrdU residues (CBB template) was annealed with various DNA primers (Figure 1A) to prepare a series of P/Ts that were chain-terminated at different positions relative to the BrdU residues. Stable +1 dNTP•RT•P/T complexes were formed by incubating each P/T with HIV-1 RT and the dNTP complementary to the template nucleotide downstream from each of the chain-terminated primers. The reaction mixture was treated with heparin to dissociate RT•P/T binary complexes. Exposure of the heparin-treated incubation mixtures to UV light resulted in formation of one predominant cross-linked product that could be separated from free DNA by SDS-PAGE and migrated at about 85 kDa, consistent with one p66 subunit of RT and one molecule of template DNA (Figure 2A). One or more fainter bands were usually observed migrating at slightly higher and lower molecular weights.

The yield of cross-linked products was dependent on the position of the primer terminus relative to the BrdU residues (Figure 2A) and on the presence of the next complementary dNTP (Figure 2B). With the CBB template, the greatest yield of cross-linked products was observed with the XP34ddG primer and dATP and, to a lesser extent, with XP33ddT and dGTP. Minimal yields were obtained with complexes formed with any of the other chain-terminated primers or with dNTPs that were not complementary to the +1 position. The most likely interpretation of these results is that the dNTP-induced conformational change brings a specific structure in RT into close proximity with one of the BrdU residues just downstream from the primer terminus. The reaction was greatly enhanced by the presence of BrdU in the template. A similar experiment carried out with CTT template showed a background level of cross-linked products, but there was no preference for cross-linking with the XP34ddG or XP33ddT primers (data not shown).

**Figure 3:**
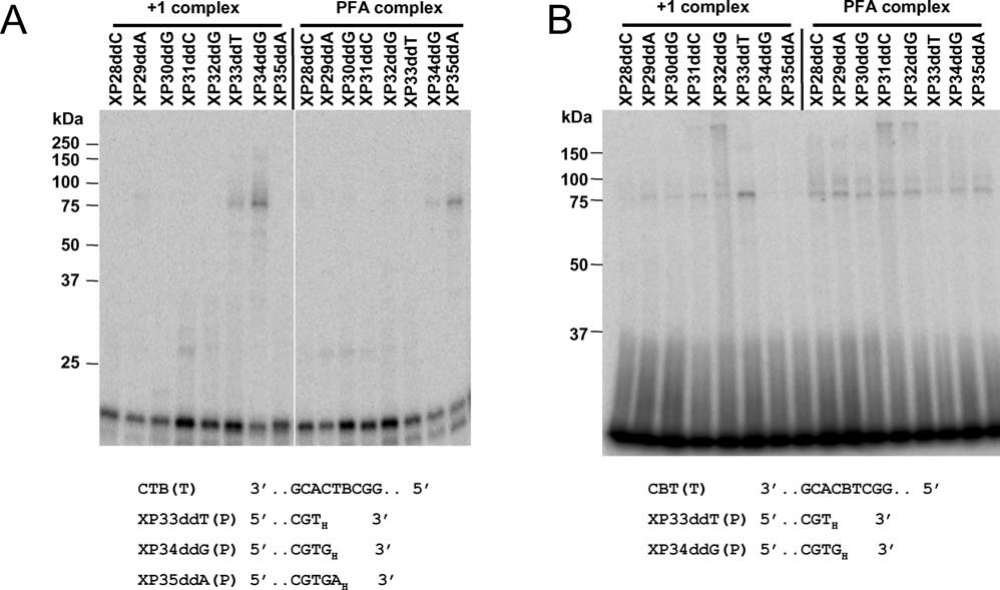
Photo-cross-linking of HIV-1 RT to P/Ts containing a single BrdU residue. Complexes in (A) were formed with 400 nM HIV-1 RT and 5 nM CTB template annealed to each of the indicated chain-terminated primers (Figure 1). Complexes in (B) were formed with 800 nM HIV-1 RT and 20 nM CBT template annealed to each of the chain-terminated primers. For both panels the reaction mixtures contained100 µM of the next complementary dNTP (+1 complex) or 3.2 mM foscarnet (PFA complex). Complexes were treated with heparin, exposed to UV light and analyzed by SDS-PAGE as in Figure 2. Positions of MW markers are shown at the left of each panel. Portions of the template and selected primer sequences are shown at the bottom of each panel.

Cross-linking experiments were also carried out with templates containing a single BrdU (CTB and CBT templates shown in Figure 1A). For CTB template, the greatest yield of cross-linked product was observed with XP34ddG primer and dATP (Figure 3A); whereas, for the CBT template, the yield was greatest with XP33ddT primer and dGTP (Figure 3B). Cross-linked products were also formed in the presence of the correct +1 dNTP (dGTP) with BrdU in the +3 position using the CTB template (Figure 3A, lane designated by the XP33ddT primer). High background reaction with the CBT template prevented us from determining whether cross-linking also occurred with the +3 position with this template (Figure 3B, lane designated by the XP32ddG primer). These results suggest that in the +1 dNTP complex RT interacts closely with the nucleobase at position +2 in the template and may also interact with the nucleotide in the +3 position.

### Cross-linking between RT and BrdU-containing templates in the presence of foscarnet

Figure 2 shows the results of cross-linking experiments with CBB template and each of the eight chain-terminated primers in the presence of 200 nM HIV-1 RT, 3.2 mM foscarnet and 2.3 mU/ml heparin. Cross-linked products were observed with foscarnet that had the same electrophoretic mobility as those observed with the +1 dNTP•RT•P/T complex. The greatest yield of cross-linked products was obtained using primer XP35ddA. Cross-linking of foscarnet complexes was also tested with templates containing single BrdU residues (Figure 3). Optimal cross-linking was seen with XP35ddA primer and CTB template (Figure 3A), but all of the primers gave similar low levels of cross-linking with CBT template (Figure 3B) in spite of the fact that both RT and P/T concentrations were elevated in this experiment. Detectable cross-linked products were observed with several of the primers; however, the levels were similar to those observed with CTT template under similar conditions (data not shown). The ability to form a specific foscarnet•RT•P/T complexes with the CBB and CTB templates coupled with the inability to detect a specific foscarnet-induced cross-link with the CBT template suggests that formation of stable foscarnet•RT•P/T complexes is dependent on sequence context.

Taken together these results show that formation of +1 and foscarnet complexes elicits close interactions between HIV-1 RT and specific positions on the template strand. This interaction was detected by cross-link formation with the first unpaired nucleotide downstream from the base pair formed by the +1 dNTP in the +1 complex and with the first unpaired base downstream from the primer terminus in the foscarnet complex. The two complexes may have similar structures; however, higher concentrations of RT were required to reliably observe specific cross-linking in the foscarnet complex and stable foscarnet complex formation depended on sequence context.

### Effect of formation of stable RT•P/T•dNTP complexes on SA interaction with biotin-containing template

**Figure 4:**
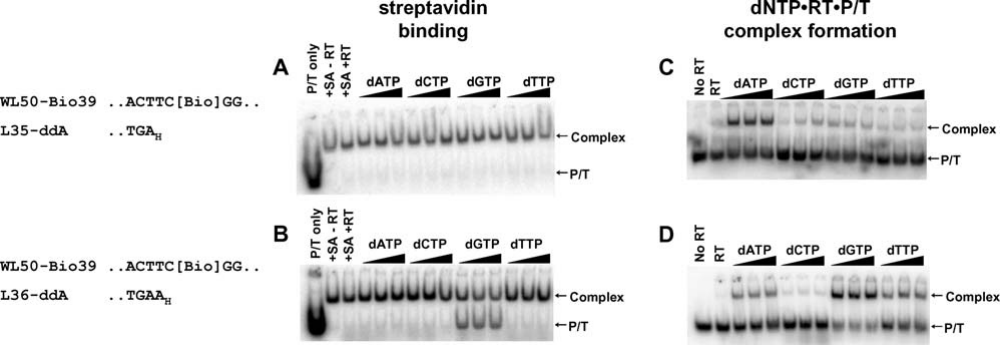
Effect of preformed RT stable ternary complexes on SA binding to a template biotin residue. Five nM 5′-^32^P-labeled P/T L35-ddA/WL50-Bio39 (A,C) or L36-ddA/WL50-Bio39 (B,D) were incubated without or with 100 nM RT in the absence of ligands or with 0.8, 3.2 or 6.4 mM dNTP. In A and B, SA (50 nM) was added for 5 min at 37°C, and 0.6 µM biotin was added to bind excess SA. Then RT was dissociated with SDS and urea, and SA-biotin-DNA complexes were separated from free DNA by electrophoresis on nondenaturing gels. For C and D, complexes formed with dNTP were transferred to ice, treated with heparin to dissociate RT•P/T binary complexes and fractionated by nondenaturing gel electrophoresis. Arrows to the right of each panel indicate positions of free DNA (P/T) and SA-biotin-DNA complexes (A,B) or dNTP•RT•P/T ternary complexes (C,D). A portion of each P/T sequence is shown. “Bio” indicates the biotin-ON linkage. Subscript “_H_” denotes a dideoxyribonucleotide residue.

The interaction between RT and the template strand was further probed by preparing DNA template oligonucleotides containing biotin at an internal position (shown in Figure 1) and assembling complexes at various positions relative to the biotin residue. SA binding was monitored by EMSA assay after treatment with urea and 0.5% SDS, which disrupts binary and ternary RT complexes but not the SA-biotin linkage (25). For P/T L35-ddA/WL50-Bio39 (Figure 4A and C) the biotin moiety was placed at position +3 with respect to the primer terminus. Binding of SA was detected in the absence or presence of each of the four dNTPs (Figure 4A). Heparin-stable RT•P/T•dNTP complexes were detected only with dATP (Figure 4C), as expected since dATP is complementary to the +1 position on the template. For P/T L36-ddA/WL50-Bio39 (Figure 4B and D) the biotin moiety was placed in the +2 position. SA binding was inhibited when dGTP (+1 dNTP) was present (Figure 4B), and dGTP-dependent formation of heparin-stable complex was evident (Figure 4D). Heparin-resistant complexes were also observed with dATP and dTTP on this P/T, although the yield was lower than with dGTP. It is possible that mismatched structures are stabilized on this P/T for unknown reasons; however, the presence of these structures did not detectably interfere with SA binding.

In summary, the presence of a +1 dNTP•RT•P/T complex did not inhibit SA binding to a template-attached biotin residue located three nucleotides downstream from the primer terminus. In contrast, formation of the +1 dNTP•RT•P/T complex did interfere with SA binding to a biotin residue located two nucleotides downstream from the primer terminus. These results are consistent with the cross-linking results and suggest that a close interaction occurred between HIV-1 RT and the +2 template base in the +1 dNTP•RT•P/T complex.

### Detection of barriers to primer extension created by SA bound to a biotin residue at a specific position in the template

**Figure 5:**
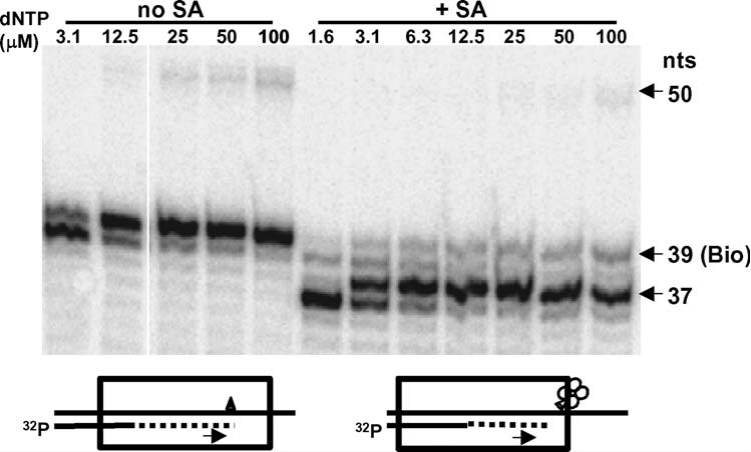
Stalling of primer extension by HIV-1 RT due to a biotin residue placed at a specific position in the template in the absence or presence of SA. (Left panel) Five nM 5′-[^32^P]-L20 primer annealed to WL50-Bio39 template was extended with 200 nM RT and various concentrations of dNTP (indicated above the lanes) and 6 µM biotin for 45 min at 37°C. (Right panel) 5′-[^32^P]-L20/WL50-Bio39 P/T was first incubated for 2-3 min with 100 nM SA at 37°C and then RT, dNTPs and biotin were added and primer extension was carried out as above. Labeled products were fractionated on 20% polyacrylamide under denaturing conditions. Arrows indicate 50 nt (full length of template), 39 nt (primer extended to the position of the biotin residue), and 37 nt (primary stop site for primer extension in the presence of SA.). Diagrams at the bottom of the figure show the direction of primer extension (arrows) and the likely position of RT (box) after stalling on a template containing the biotin residue (triangle) in the absence (left) or presence (right) of SA (shown as a tetramer of circles).

In the absence of SA, RT extended a DNA primer to the site of the biotin residue in the DNA template oligonucleotide WL50-Bio39 and added a base across from the biotin but most of the DNA molecules were not extended further (Figure 5, left panel). In the presence of SA, primer extension stopped three nucleotides upstream from the biotin residues when the dNTP concentration was low and two nucleotides upstream in the presence of elevated dNTP concentrations (Figure 5, right panel). These results indicate that the presence of bound SA prevented further primer extension, which may be due to steric collision between RT and the attached SA, or bound SA may introduce a conformational change into the P/T that prevents RT from moving forward.

### Detection of barriers created by bound SA to digestion of the template strand with RecJ_F_

**Figure 6:**
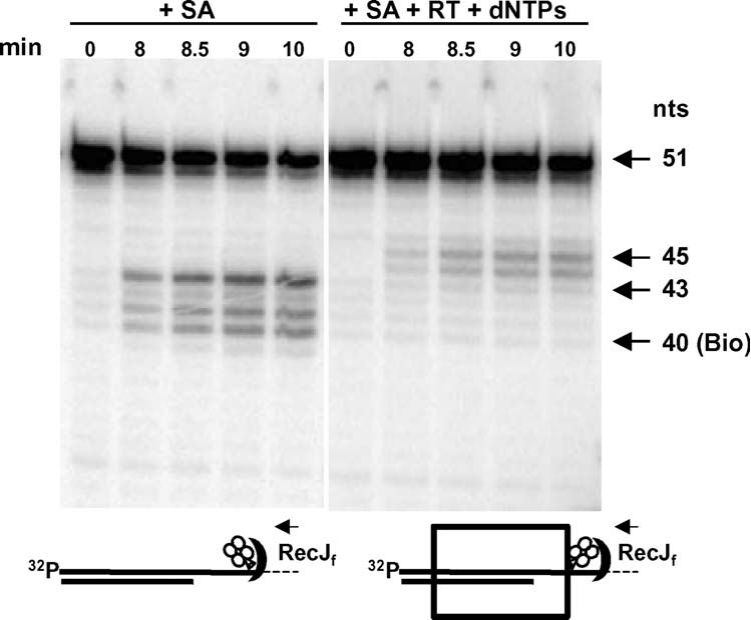
Products of template digestion by RecJ_F_ exonuclease in a P/T containing a bound SA molecule in the absence or presence of HIV-1 RT and dNTPs. (Left panel) Five nM L32 primer was annealed to 3′-[^32^P]ddAMP-labeled WL50-Bio39 template and incubated with 50 nM SA at 37°C for 5 min followed by incubation with RecJ_F_ (1 U per µl of reaction mixture) at 37°C for the indicated times. (Right panel) L32 primer/3′-[^32^P]ddAMP-WL50-Bio39 template was incubated with SA as described above. Then, 12.5 nM HIV-1 RT and 100 µM each of the four dNTPs were added and incubated at 37°C for 10 min followed by digestion with RecJ_F_ as above. Digestion products were fractionated on 20% polyacrylamide under denaturing conditions. Arrows at the right of the panels indicate 51 nt (full-length labeled template) and major pause sites for RecJ_F_ digestion at 45 nt and 43 nt. A digestion product stopping at the biotin residue would be 40 nt, designated 40(Bio). Diagrams at the bottom show the position of RecJ_F_ (filled crescent) after digestion reaches a pause site in the absence (left) or presence (right) of RT (box) and dNTPs. Arrows indicate direction of RecJ_F_ digestion. SA is shown as a tetramer of circles and biotin as a triangle.

We previously showed that RecJ_F_ digestion from the 5′ end of the template could be used to map the downstream border of the stable complexes of RT with primer-template [8]. In the absence of RT, SA bound to WL50-Bio39 template served as a barrier to RecJ_F_ producing a cluster of digestion products beginning 3 nucleotides from the biotin and extending to the position of the biotin residue itself (Figure 6, left panel). By contrast, in the presence of RT and 100 µM dNTPs, RecJ_F_ digestion was blocked at positions four and five nucleotides downstream from the biotin residue (Figure 6, right panel). The structural changes that account for the introduction of these new downstream barriers are not clear since RT, SA and the nucleic acid substrate may undergo distortions or sideways displacement as RT collides with SA, but the results are consistent with the biotin-bound SA molecule being pushed forward by RT, displacing the barrier to RecJ_F_ digestion forward by approximately two nucleotides.

## Discussion

We have shown that formation of the +1 dNTP•RT•P/T complex is characterized by a strong interaction between HIV-1 RT and the downstream DNA template strand. Several lines of evidence indicate that the primary interaction site is the +2 position on the template. (a) UV-induced cross-linking between RT and the DNA template strand in the +1 dNTP•RT•P/T complex was most efficient when BrdU was placed in the +2 position on the template. (b) SA binding to a biotin residue in the +2 position on the template was inhibited when +1 dNTP•RT•P/T complex was formed; whereas, binding to biotin in the +3 position was not affected. (c) SA pre-bound to a biotin residue in the template strand caused RT to stall two nucleotides upstream from the biotin residue suggesting that RT encountered a barrier created by the bound SA approximately two nucleotides beyond the position of the last nucleotide incorporated. Finally, (d) a barrier to exonuclease RecJ_F_ created by SA bound to a template biotin residue was shifted forward approximately two nucleotides as a result of RT pushing against the barrier from the upstream side. This also suggests that the stalled RT protrudes downstream approximately two nucleotides beyond the position of the last nucleotide incorporated. Our results agree with previous reports. Dash et al. [18] have reported that nucleotide analogs containing a conformationally locked cyclohexane ring in place of the pentose sugar create a barrier to primer extension causing RT to stall two nucleotides upstream with minor stalling observed 3 and 4 nucleotides upstream. In addition, Winshell and Champoux [17] have shown that a complementary DNA oligonucleotide placed downstream from the primer terminus is melted by formation of +1 dNTP•RT•P/T complex, with the melted region extending two base pairs in front of the primer terminus.

We have employed a unique combination of techniques to investigate the most significant interactions involved in forming stable complexes between RT and P/T. This approach may be of general use in elucidating the role of specific interactions in functional complexes between polymerases and their nucleic acid substrates. Because we have focussed on stable complexes, we did not detect weaker contacts that may be important for binary complex formation and may contribute to RT activity. The existence of such downstream interactions has been suggested by several previous studies. Patel et al. [26] reported that P/T with a template overhang of 6 nucleotides bound to RT with a *K*_d_ of 8 nM, which increased 4-fold to 32 nM when the template overhang was reduced to 3 nucleotides. Peletskaya et al. [27], [28] showed that a template extension of 5 to 7 nucleotides was required for maximal efficiency of cross-linking between sulfhydryl groups tethered at specific positions in the template strand and cysteine residues introduced into the fingers subdomain of RT. This cross-linking was associated with the open binary complex and was suppressed by the presence of +1 dNTP. These results suggest that downstream interactions are important for binary complex formation; however, altered binary complex stability is likely to have implications for the subsequent binding of dNTPs. Boyer and colleagues [16] showed that a template extension of at least 3 to 4 nucleotides was needed to confer sensitivity to dideoxyinosine or dideoxyadenosine triphosphate nucleotides on wild type HIV-1 RT implying that downstream interactions play a role in nucleotide discrimination. In addition, formation of +1 dNTP•RT•P/T ternary complexes is impaired when the template overhang is three or fewer nucleotides [3], [15].

Binary and ternary complexes may differ in the position as well as the strength of interactions needed to maintain their structure. Binary complex may be stabilized by relatively weak interactions over an extended region of the primer-template that allow the enzyme to move between positions and yet hold the complex together during the cycling process that gives rise to processive DNA synthesis. Ternary complexes require strong interactions that hold the enzyme in a specific position. Because of the presence of these stronger interactions, we are unable to determine whether the weaker interactions also exist in the ternary complexes but are masked in our experiments or whether the conformational changes associated with ternary complex formation lead to reorganization of the structure so that the weak downstream interactions are no longer present.

Enzymatic footprinting experiments that show protection of the template 7 to 9 nucleotides beyond the primer terminus [2], [8], [17] are often interpreted as evidence for downstream interactions; however, intrinsic structural features of the endonucleases or exonucleases used in mapping experiments limit the extent of digestion causing the resulting footprint to appear larger than the complex being mapped. Our results suggest that RecJ_F_ can only approach within 4 or 5 nucleotides of the biotin residue containing bound SA when RT is pushing from the other side (Figure 6). For bacteriophage lambda exonuclease, further digestion is blocked at distance of about 11 nucleotides from the bound SA (data not shown), which is consistent with structural evidence showing that the catalytic residues are located at an internal site in this enzyme [29]. DNase I and nuclease SI, which have been used to produce downstream footprints of RT on the template strand [2], [17], are also likely to have steric shadows that cause the footprints to extend several nucleotides beyond the true borders of the complex as has been previously noted [17]. Chemical footprinting is much less susceptible to steric effects, and hydroxyl radical cleavage of the +1 dNTP•RT•P/T complex shows protection extending only three nucleotides downstream from the primer terminus [30]. In summary, footprinting data on the ternary complexes are consistent with close contact between RT and the first two or three nucleotides of the template extending beyond the primer terminus.

Three residues of the downstream template overhang are resolved in the crystal structure of the +1 dNTP•RT•P/T ternary complex (PDB databank, 1RTD)[4]. R78 contacts the phosphate between positions –1 and +1, and W24 contacts the phosphate between +2 and +3. Additional contacts with the +1 nucleotide are made by L74, V75, D76 and G152. F61 interacts both with nucleotide +1 (via the deoxyribose moiety) and +2 (with the nucleobase). Up to four residues are resolved in a second +1 dNTP•RT•P/T ternary complex (PDB data bank,1ROA) [Tuske et al., unpublished, discussed in Ref. 28]; however, the path of the template extension differs in this structure from that in the 1RTD structure. A different path was observed for the template extension in binary complex structures with RT[5].

The residues in RT that are cross-linked with template BrdU in our studies have not been identified. In UV photo-cross-linking studies carried out with Klenow fragment of *E. coli* DNA polymerase (KF), Y771 was identified as the major cross-linking target in ternary complexes with BrdU at the +2 or +3 template position [31] or with a TT sequence at positions +2 and +3 in the template [32]. A comparison of residues homologous to Y771 in crystal structures containing Bst DNA polymerase [33] or Klentaq DNA polymerase [34] with the crystal structure of the HIV-1 RT ternary complex strongly suggests that F61 in RT plays a structural role analogous to that of the Y771 residue of KF. Turner et al. [31] also observed cross-links formed with Y766 of KF DNA polymerase when BrdU was in the +1 position; however, in contrast to Y771, the efficiency of cross-linking at the +1 position was greater when the UV treatment was applied to the binary complex and was inhibited by ternary complex formation. These observations agree with crystallographic studies on prokaryotic Pol 1 class enzymes showing that residues homologous to Y766 are stacked on the –1 template base in the binary complex and repositioned to the side of the helix upon formation of the ternary complex [34], [35]. No residue has been identified in HIV-1 RT that plays a role similar to that of Y766 in KF.

Mutants of F61 in HIV-1 RT have been studied extensively to determine the functional roles of this residue. F61 mutations have been shown to increase fidelity of nucleotide incorporation [36], [37], reduce the ability of RT to extend a mismatched primer terminus [36], decrease processivity [37], [38], either decrease or increase strand displacement activity [38], [39], and enhance the ability to bypass lesions in the template strand [18]. These results are consistent with a major role for F61 in RT function mediated through interactions with the template overhang.

The foscarnet•RT•P/T complex formed cross-links with the template most efficiently when BrdU was in the +1 position. This reflects the fact that the complex is positioned one nucleotide upstream relative to the +1 dNTP complex as previously reported [7], [8]. In contrast to results with the +1 dNTP•RT•P/T complex, efficient cross-linking of the foscarnet complex required high concentrations of foscarnet and varied with template sequence context. It is possible that the foscarnet and +1 dNTP ternary complexes are structurally similar and that the smaller ligand contributes fewer contacts resulting in a complex with reduced stability; however, the complex formed by RT in the untranslocated position may be intrinsically less stable and other factors may contribute to the differences between these complexes.

We have observed tight interactions between RT and the +2 nucleotide on the template in the +1 dNTP•RT•P/T stable complex that may serve as a “lock” to hold RT in place during phosphodiester bond formation. These interactions may also serve as a wedge to displace a complementary oligonucleotide fragment bound to the template in front of the growing DNA chain that may impede chain elongation. Interactions between RT and the template at positions further downstream were not observed and may not be required to maintain the integrity of stable ternary complexes.
